# Platelet extravasation in the microvasculature: an under-appreciated role for platelets

**DOI:** 10.3389/fimmu.2026.1764398

**Published:** 2026-05-18

**Authors:** Justin A. Courson, Vahid Afshar-Kharghan, Alan R. Burns, Rolando E. Rumbaut

**Affiliations:** 1 Center for Translational Research on Inflammatory Diseases, Michael E. DeBakey Veterans Affairs Medical Center, Houston, TX, United States; 2 Department of Medicine, Baylor College of Medicine, Houston, TX, United States; 3 MD Anderson Cancer Center, University of Texas, Houston, TX, United States; 4 College of Optometry, University of Houston, Houston, TX, United States

**Keywords:** platelets, extravascular, platelet extravasation, microvasculature, transmigration, transcytosis, inflammation, parenchyma

## Abstract

In recent years, evidence has accumulated highlighting the presence and role of platelet extravasation – wherein platelets accumulate in tissue parenchyma – at a variety of sites throughout the body. While platelets are traditionally known for their roles in hemostasis and thrombosis, it is evident that platelets are potent mediators of inflammation across an array of physiological and pathological contexts. While anucleate and small in size, platelets contain a rich diversity of molecules in their granules capable of modulating cell proliferation, tissue repair, and a host of immune responses. There is growing evidence that platelet extravasation out of the vascular space and into tissue parenchyma not only occurs in a host of pathological conditions, but it may also play a role in inflammation and disease progression. This review examines the evidence of platelet extravasation in a number of microvascular beds, including the skin, tumor, cortical, corneal, lung, and liver microvasculature, discussing mechanisms of extravasation and roles platelets play in these contexts. Understanding the dynamics and functional relevance of platelet extravasation may provide insight into novel therapeutic targets and lines of scientific inquiry into diseases and conditions affecting the microvasculature.

## 1 Introduction

The role of platelets has been traditionally limited to the cessation of bleeding following vascular injury (hemostasis) and the pathological formation of blood clots (thrombosis) (1). However, it is now evident that platelets play an essential role as mediators of inflammation in a broad range of conditions in various microvascular beds and pathologies (2–5). Platelets are now widely appreciated for their multifaceted capabilities, participating in such varied activities as the response to microbial infection and viral clearance, recruitment of innate immune cells and modulation of their responses, regulation of antigen presentation and enhancement of the adaptive immune response, allergic lung inflammation, sterile liver injury and repair, atherosclerosis, and cancer progression (6–12). As such, there is a growing awareness of platelets as immune cells. While platelets are traditionally defined as intravascular mediators of hemostasis and thrombosis, accumulating evidence suggests that their functional activities extend beyond the confines of the vasculature.

**Figure 1:**
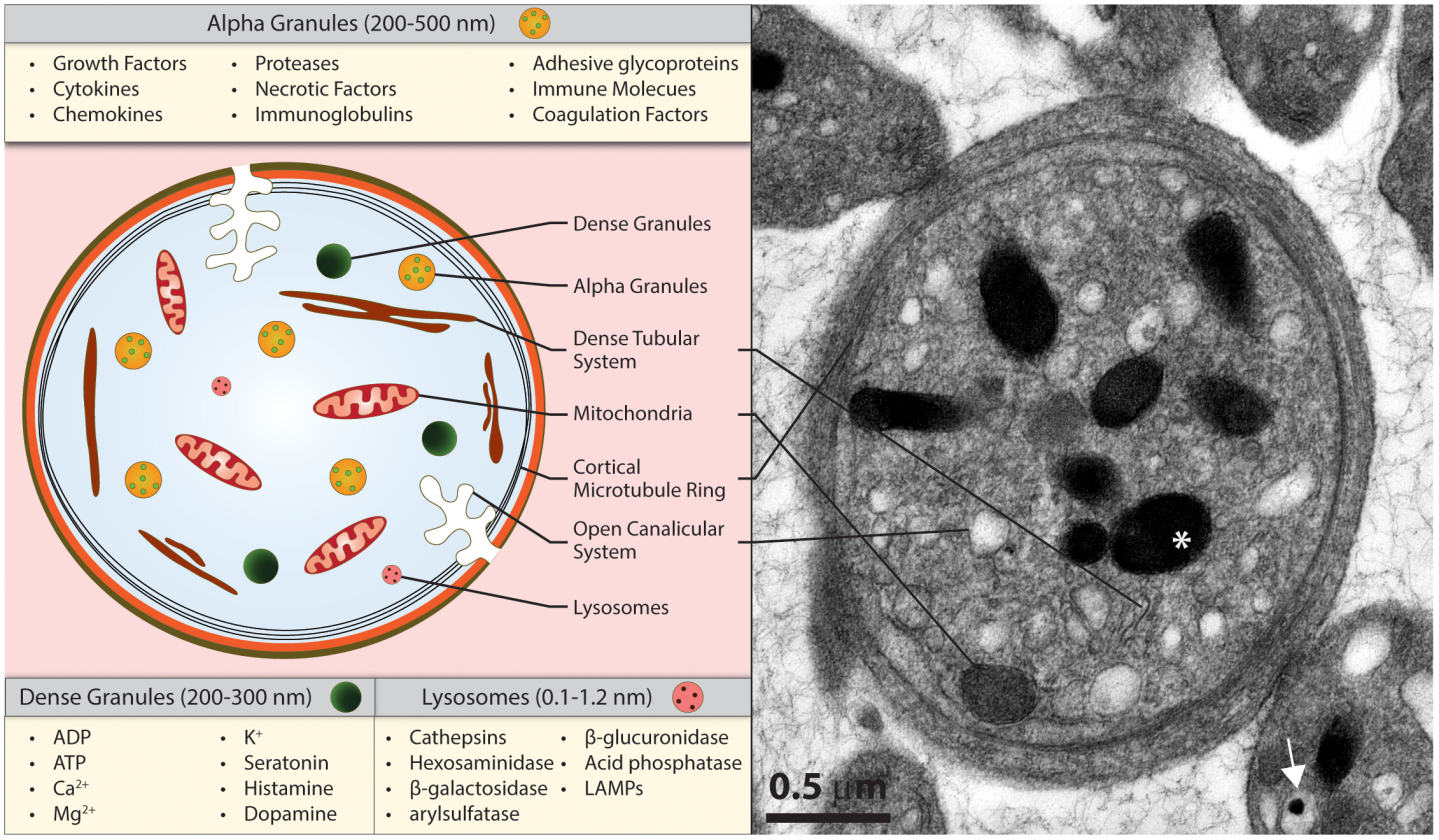
Platelets contain a diverse array of granules with potent intravascular and extravascular effects. While platelets do not contain a nucleus, they maintain a limited capacity for protein synthesis translated from mRNA found within the cell. Alongside α-granules, dense granules, and lysosomes, platelets contain a dense tubular system, an open canalicular system, mitochondria, and a cortical ring of microtubules. Scale bar = 0.5 µm. *denotes an alpha granule, while the white arrow denotes a dense granule.

Human platelets are small anucleate cells, with an average diameter of 1.5 to 3 µm, thickness of ~0.5 µm and volume of ~8 femtoliters, each platelet contains between 50 and 100 granules housing more than 300 unique soluble proteins (13). Platelet granules are differentially distributed and released in response to a variety of stimuli (14) (**Figure 1**). Platelet α-granules are the most abundant (~50–80 per cell) and contain growth factors, proteases, immunoglobulins, and adhesive glycoproteins, along with a large number of cytokines and chemokines capable of affecting activities ranging from leukocyte recruitment and adhesion to cell proliferation and differentiation (15). The contents of dense granules, which are approximately 10-fold fewer in number than α-granules, can influence leukocyte recruitment and function alongside endothelial cell modulation, and lysosomal granules (typically 1–2 per cell) containing factors that support activities such as extracellular matrix degradation, fibrinolysis, and receptor cleavage (16). Through the release of cytokines, chemokines, and growth factors stored within their granules, platelets are adept modulators of the local microenvironment, influencing the progression and resolution of inflammation in a wide array of tissues and organs. In addition to their granules, platelets contain messenger RNA, a fairly robust proteasome complete with antigen-presenting machinery, mitochondria, a dense tubular system similar to the endoplasmic reticulum, a cortical ring of microtubules important in maintaining the shape of non-activated platelets, as well as an open canalicular system capable of capturing and storing plasma proteins (17). Thus, platelets are capable of affecting a host of physiological and pathological processes. Given their broad range of capabilities, it is important to note that platelets have been observed beyond the endothelial barrier in a diversity of inflammatory, malignant, infectious, and fibrotic conditions, aggregating within tissue parenchyma and in close proximity to immune and stromal cells. While much attention has focused on the role of platelets within the vasculature, there is increasing evidence of a role for extravasated platelets in a number of vascular beds throughout the body.

Platelets play a necessary role in hemostasis and thrombosis and contain a number of receptors that participate in their activation and support the interaction between platelets and proteins at sites of vascular injury. When the integrity of the vascular endothelium is compromised, the underlying subendothelial matrix becomes exposed to the vascular lumen, setting in motion a series of events aimed at repairing the damaged vasculature. Following vascular injury, platelets become activated in response to their interaction with proteins such as von Willebrand Factor (VWF) and collagen, or with soluble agonists such as ADP, thrombin, and thromboxane A2 (18). In the presence of the serine protease thrombin, which is released at sites of vascular injury, platelet PAR1 (PAR3 in mice) and PAR4 allow platelet activation in proximity to the site of damage (19, 20). Interaction between platelet GPIb-IX-V (a complex including GPIα, GPIbβ, GPIX and GPV) and VWF, which becomes bound to collagen at sites of injury, allows platelets to roll on and adhere to the vascular wall. Following this, the platelet receptors glycoprotein VI (GPVI) and α2β1 integrin mediate platelet interaction with collagen, allowing for firm arrest of platelets on the vessel wall. Interactions between GPIbα and VWF result in the presentation and activation of αIIbβ3 integrin, allowing for the binding of αIIbβ3 with soluble fibrinogen resulting in stable platelet-platelet aggregation and the formation of a fibrin clot (21). The binding of αIIbβ3 to its ligand results in platelet spreading, the secretion of granules, stabilization of platelet adhesion and platelet-platelet aggregation, and ultimately clot retraction (18).

**Figure 2:**
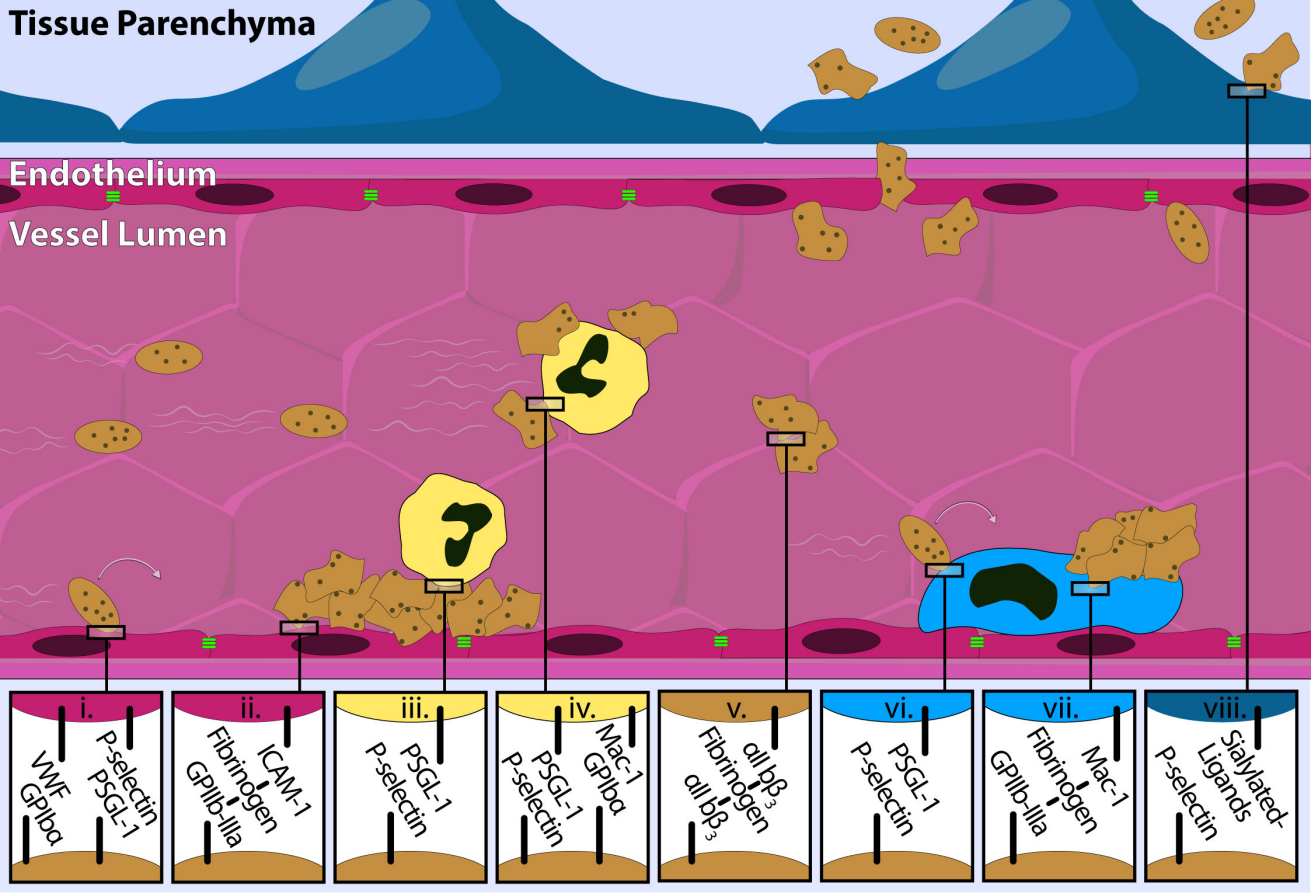
Platelet-cell dynamics are mediated by well-regulated protein-protein interactions. Platelets (brown) engage in a wide variety of cell-cell interactions requiring a host of well-regulated surface proteins. Interactions with endothelial cells (pink; associated junctional proteins in green), leukocytes (yellow), monocytes (light blue) and parenchymal cells (dark blue) all require coordinated surface protein interactions that are vital to the platelet response to injury. Example interactions shown include platelet rolling (i.) and arrest (ii.) on vascular endothelium; platelet-leukocyte aggregation (iii. & iv.); platelet-platelet aggregation (v.); platelet tethering to endothelium-bound monocytes (vi.) and subsequent stable platelet adhesion (vii.); and platelet adhesion to parenchymal cells (e.g., astrocytic end feet) (viii.).

While platelets are primarily viewed as mediators of hemostasis and thrombosis, their interactions with immune cells and vascular endothelium have been extensively described in the literature. Receptors on platelets such as PAR1, PAR4, P2Y1, PSGL-1, P2Y12, TP, GPVI, GPIb-IX-V Complex, P-selectin, GPIbα, ICAM-1, ICAM-2, CD40L, CLEC-2, and a number of integrins including αIIbβ2, αVβ3, αVβ1, and α6β1 allow platelets to interact with immune cells, coagulation factors, and the vascular endothelium(**Figure 2**) (22). Activated platelets can interact with leukocytes, such as neutrophils and monocytes, through mechanisms including the binding of P-selectin to PSGL-1, GPIbα binding to Mac-1, as well as the binding of integrin αIIbβ3 to fibrinogen which in turn binds to leukocyte β2 integrins (23, 24). In addition, activated platelets express CD40L, a membrane glycoprotein in the tumor necrosis factor family of molecules, which can interact with CD40 on endothelial cells, resulting in an inflammatory response (25). The interactions between platelets and other immune cells have been extensively reviewed (22). The primary purpose of this review is to highlight platelet extravasation and focus on the mechanisms and known functional consequences of platelet extravasation. A unified understanding of platelet extravasation as a functional mechanism in health and disease has important implications for inflammatory regulation, tumor biology, vascular permeability, alongside therapeutic targeting. Defining the molecular mechanisms governing platelet extravasation and retention in tissues, and exploring the dynamics of platelet extravasation across disease paradigms has the potential to reveal novel points of intervention and therapeutic strategies.

## 2 Platelet extravasation

Studies evaluating the physiological and pathological role of platelets have noted the presence of extravasated platelets in tissue parenchyma (26–33). Platelet extravasation is an often underrecognized and poorly understood phenomenon that occurs in several vascular beds and in response to various diseases and disorders. While the precise mechanisms of platelet extravasation remain to be fully defined, there is evidence to suggest that it may involve a complex interplay between platelets, endothelial cells, neutrophils, other immune cells, and parenchymal cells within the tissue microenvironment. Studies have revealed instances of platelet extravasation in several contexts, challenging the traditional notions of platelet behavior. This review will outline the evidence of platelet extravasation in a number of vascular beds, including the dermal, tumor, cortical, corneal, lung, and liver microvasculature, as well as discuss the known roles of platelets in the parenchyma and mechanisms by which platelets extravasate from the microvasculature. Each of these vascular beds presents unique challenges and opportunities for understanding the mechanisms and functional significance of platelet extravasation.

### 2.1 Dermal inflammation

**Figure 3:**
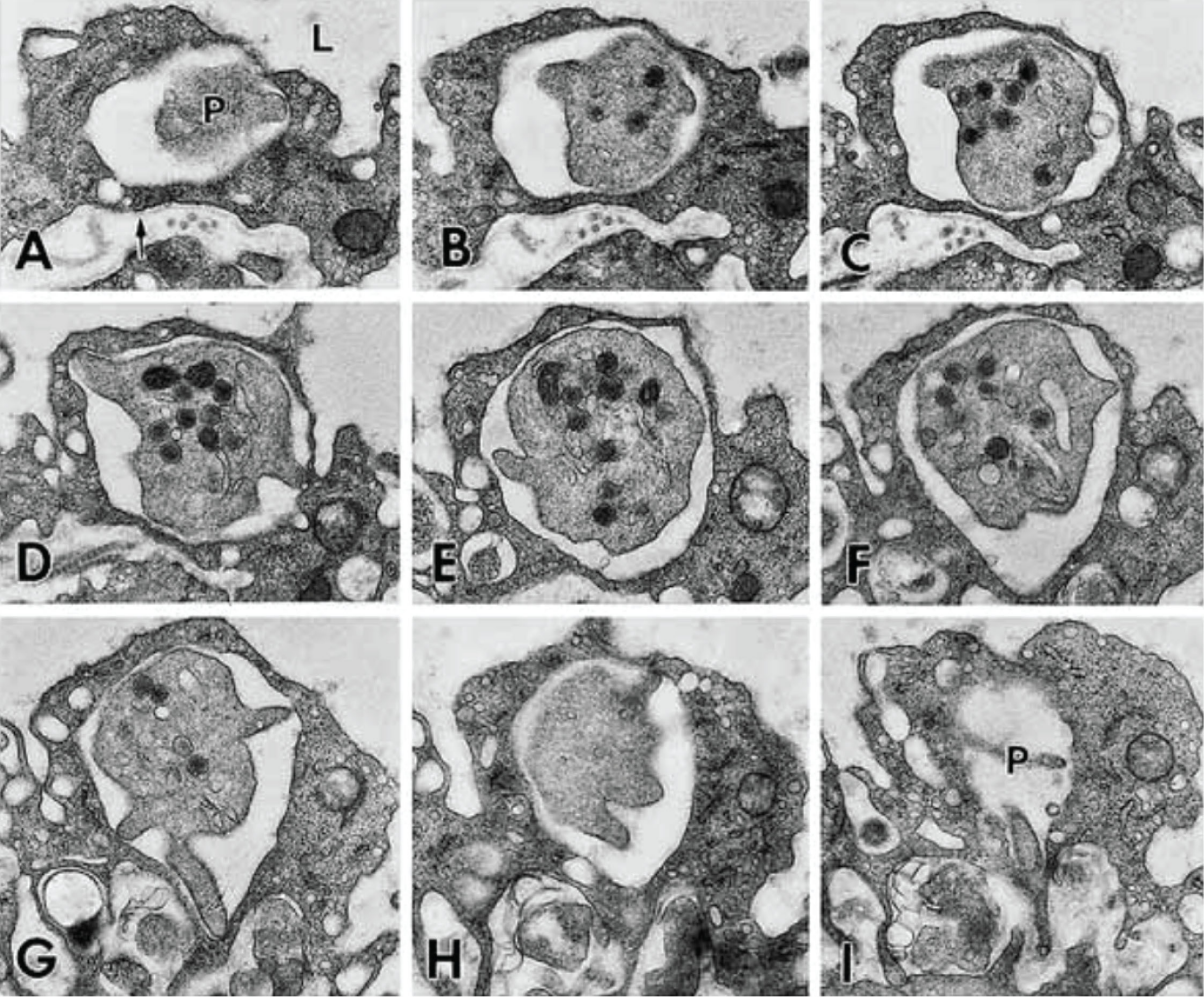
Transcellular trafficking of platelets through the vascular endothelium in acute dermal inflammation. Serial electron micrographs illustrate a platelet undergoing transcellular extravasation through venular endothelial cells **(A–I)**. Section numbers 2, 4, 6, 8, 10, 14, 17, 20, and 22 in a single series of transmission electron microscopy images containing 23 consecutive serial sections are represented. One end of the platelet (P) is seen in panel **(A)**, which then expands across the remaining sections to reveal alpha granules and platelet organelles. A final platelet process (P) is seen in panel **(I)**. Extravasating platelets show minor shape changes but do not exhibit stereotypical ultrastructural features related to activation and degranulation. Platelets were found to be completely enclosed within endothelial vacuoles, and endothelial cell junctions were intact. L, lumen; arrow, basal lamina. Imaging magnification = x16,500. Reproduced with permission from Feng et al., *Int Arch Allergy Immunol* 1998;116:188-195. ^©^ 1998 S. Karger AG, Basel (26).

Platelet extravasation into the parenchymal space in the skin has been shown to occur in the absence of direct vascular trauma. One of the earliest studies in which platelets were detected extravasating in the absence of direct endothelial damage was conducted in the skin microcirculation (26). Feng et al. demonstrated in 1998 that within 15 minutes of intradermal injection of 10–^5^ M N-formyl-methionyl-leucyl-phenylalanine, a model for dermal inflammation, platelets begin to extravasate through the vascular endothelium in guinea pig flank skin. Using consecutive serial electron micrographs, the authors demonstrated conclusively that platelets crossed undamaged venular endothelium. While platelets initially adhered to the luminal surface of the vascular endothelium, some of them continued to pass through the endothelial cells before crossing the vascular basal lamina and entering the tissue parenchyma. Using serial sections, the authors also showed that platelets exited venules utilizing endothelial cell cytoplasmic vacuoles (**Figure 3**), while inter-endothelial cell junctions remained closed. Platelet-containing vacuoles were located near the inter-endothelial cell borders, in a region of endothelial cell cytoplasm that is rich in vesiculo-vacuolar organelles (VVOs) involved in the transcellular trafficking of plasma solutes (34). Interestingly, in the majority of cases, these platelets underwent extravasation with little to no extensive evidence of activation and granule release, in contrast to what occurs during platelet accumulation at sites of endothelial cell damage. In 2022, Currie et al. published additional evidence of platelet extravasation in the dermis using a dorsal skin-fold model of immune complex-mediated vasculitis (35). In that study, the authors showed that platelet recruitment began approximately 30 minutes following vasculitis induction and was only observable at points where neutrophil diapedesis had occurred. Furthermore, platelet extravasation in this model was dependent upon glycoprotein VI (GPVI) and C-type-lectin-like receptor 2 (CLEC-2). Similar to the findings in the 1998 manuscript by Feng and colleagues, despite the presence of vascular inflammation, this group noted that extravasated platelets maintained intact intracellular granules. It has been further demonstrated in GPVI and CLEC-2 double-knockout mice that platelet extravasation is enhanced during the initial inflammatory phase (1–4 days post-injury) and is associated with heightened angiogenesis and diminished inflammation, followed by accelerated wound closure during the first 5 days of healing (36). Similar results were found using both topical and low-dose dasatinib treatment, a downstream signaling inhibitor of GPVI and CLEC-2 (37, 38).

The transport of platelets into the extravascular space via transcytosis is a distinct and highly regulated process, separate from the leakage of platelets that occurs as a result of bleeding or vascular damage. Furthermore, the population of extravascular platelets differs in that most of the aforementioned platelets do not appear to degranulate in the process of leaving the vasculature. As such, they may represent a sub-population of platelets that play a distinct role in the extravascular space separate from the role of platelets in direct tissue injury or vascular trauma. While the functional role of extravascular platelets in the skin is unclear, it has been theorized that platelets have additional extravascular functions, particularly in the context of inflammation. The extravascular release of platelet granule proteins may have roles in extravascular coagulation, parenchymal cell polarization, and vascular tone and permeability (26). Further insight into the mechanisms of platelet extravasation in the context of dermal inflammation could provide valuable information about immune responses and tissue repair in dermatological disorders.

### 2.2 Malignant tumor microvasculature

The tumor microenvironment is made complex by the presence of cancer cells, immune cells, and mesenchymal cells, among others (39). Among these, it has been reported that platelets play a role in cancer progression and metastasis. Platelets have been proposed to support cancer angiogenesis and metastasis, with thrombocytopenia being associated with a significant reduction in the number of tumor metastases, and elevated platelet counts being associated with tumor metastasis and poor prognosis (40). Platelets induce endothelial cell proliferation and subsequent angiogenesis, which is required for tumor growth and vessel formation in the tumor microenvironment. Platelet α-granule release in response to hypoxia can promote the recruitment of bone marrow-derived cells, which contribute to blood vessel maturation, further underscoring the importance of platelets in tumor angiogenesis (41). Following tumor cell intravasation, tumor cell-induced platelet aggregation occurs within the vasculature, which acts to protect, or cloak, tumor cells from the immune system and subsequent clearance (42). Platelets also facilitate tumor cell contact and arrest on vascular endothelium, as well as the processes of endothelial cell retraction and tumor cell extravasation, which are necessary for tumor metastasis (43).

**Figure 4:**
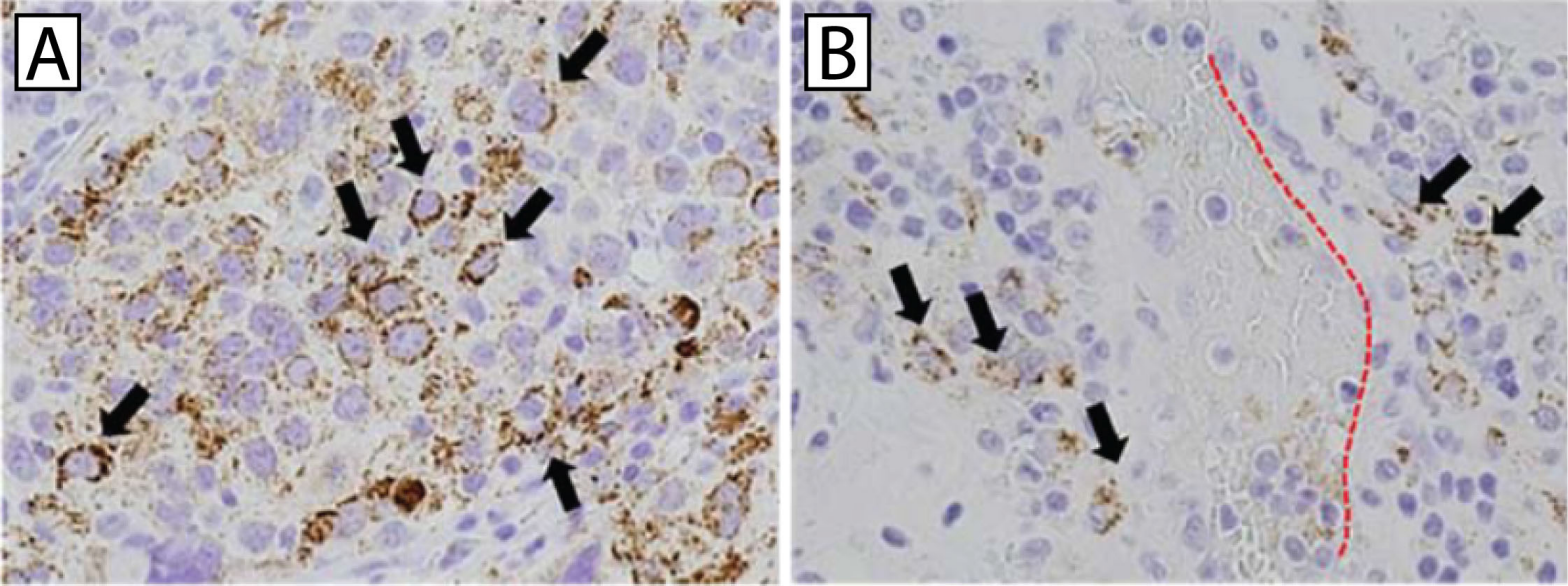
Platelets invade the tumor microenvironment and promote metastasis. Immunohistochemical staining of CD42b in primary breast cancer biopsy specimens. **(A)** CD42b+ platelets (arrows) can be seen decorating primary tumor cells. **(B)** Tumor cells decorated with CD42b+ platelets (arrows) are visible in the perivascular tissue (dotted red line delineates the blood vessel). Investigators also found platelet-decorated tumor cells in the immediate vicinity of capillaries as well as within blood vessels. Adapted with permission from Ishikawa et al., *Oncol Rep* 2016;36:787-794. ^©^ Spandidos Publications (12).

Recently, platelet extravasation into the primary tumor site has been associated with tumor growth, cancer progression, and an overall poor prognosis in models of ovarian cancer, hepatocellular carcinoma, pancreatic cancer, breast cancer, and gastric cancer (12, 30, 40, 44, 45). Miyashita et al. reported aggregated platelets in the epithelial-mesenchymal transition region at the invasive front of pancreatic tumors (40). They found CD42b expression primarily in the pericellular space surrounding cancer cells, and that cancer cells adherent to platelets invade blood vessels in the tumor microenvironment. They also reported a correlation between CD42b expression levels and loss of E-cadherin expression. Loss of E-cadherin is associated with increased tumor cell invasiveness, metastasis, and poor prognosis in a number of solid tumor cancers, including pancreatic cancer (46, 47). While platelet extravasation has been linked to chemoresistance, activation of extravasated platelets in the tumor microenvironment has been demonstrated to promote tumor cell migration into the vasculature and metastasis (12, 40) (**Figure 4**). In breast cancer, the presence of platelets in the primary tumor is associated with a reduced response to neoadjuvant chemotherapy, and in gastric cancer, it has been correlated with resistance to treatment (12, 44).

**Figure 5:**
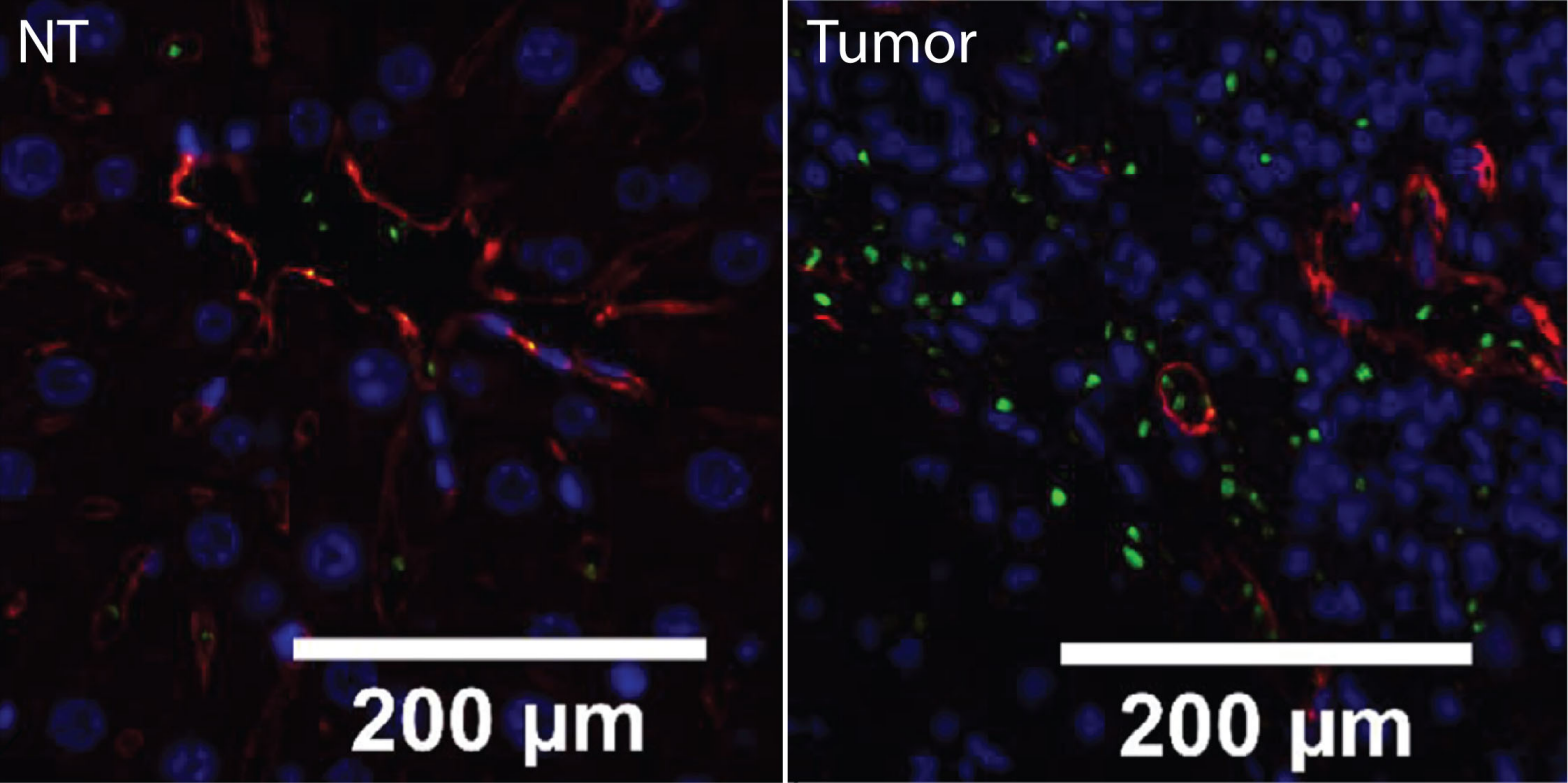
Platelets extravasate into the tumor microenvironment in a CX3CL1-CX3CR1-dependent manner. Hepatocellular carcinoma liver sample and adjacent paired non-tumorous tissue. Platelets are labeled with anti-CD41 (green), vascular endothelial cells with anti-CD31 (red), and nuclei with DAPI (blue). Extravasated platelets are visible in the extravascular space in hepatocellular carcinoma tissue, where no extravascular platelets can be seen in non-tumorous tissue. The number of extravascular platelets was found to be significantly increased (p < 0.001) in hepatocellular carcinoma samples. Adapted from Miao et al., *Mol Oncol* 2020;14:2546-2559. CC BY 4.0. Changes were made (30).

Despite reports of platelet extravasation in the primary tumor microenvironment, the role of extravasated platelets is still relatively unknown. While platelets are often considered to be non-motile, Miao et al. found that hypoxia induced platelet migration via upregulation of CX3-C chemokine ligand 1 (CX3CL1) expression (30). In this study, the release of CX3CL1 by hepatocellular carcinoma cells directly induces platelet migration in both *in vitro* and *in vivo* scenarios, and inhibition of CX3CR1 in platelets blocks platelet extravasation (**Figure 5**). Blocking Syk and PI3K significantly reduces platelet migration as well. In this study, platelets extravasate into the tissue parenchyma in human hepatocellular carcinoma tissues, but not into adjacent tumor-free tissues. Furthermore, hypoxia was demonstrated to enhance platelet migration via upregulation of CX3CL1 expression. Interestingly, extravasated platelets promote hepatocellular carcinoma cell apoptosis (30). Moreover, platelet extravasation is detected in mouse and human hepatocellular carcinoma tissues. These findings may extend to other cancers, as CX3CL1 is known to induce immune cell migration and is expressed in cancers such as breast cancer, glioblastoma, and ovarian cancer (48–50). Of note, however, cancer therapies such as oxaliplatin chemotherapy treatment increase platelet extravasation into the liver and are associated with hepatic sinusoidal obstruction syndrome (31, 51). As such, the potential adverse effect of cancer treatments targeting platelet extravasation may be an important therapeutic consideration.

**Figure 6:**
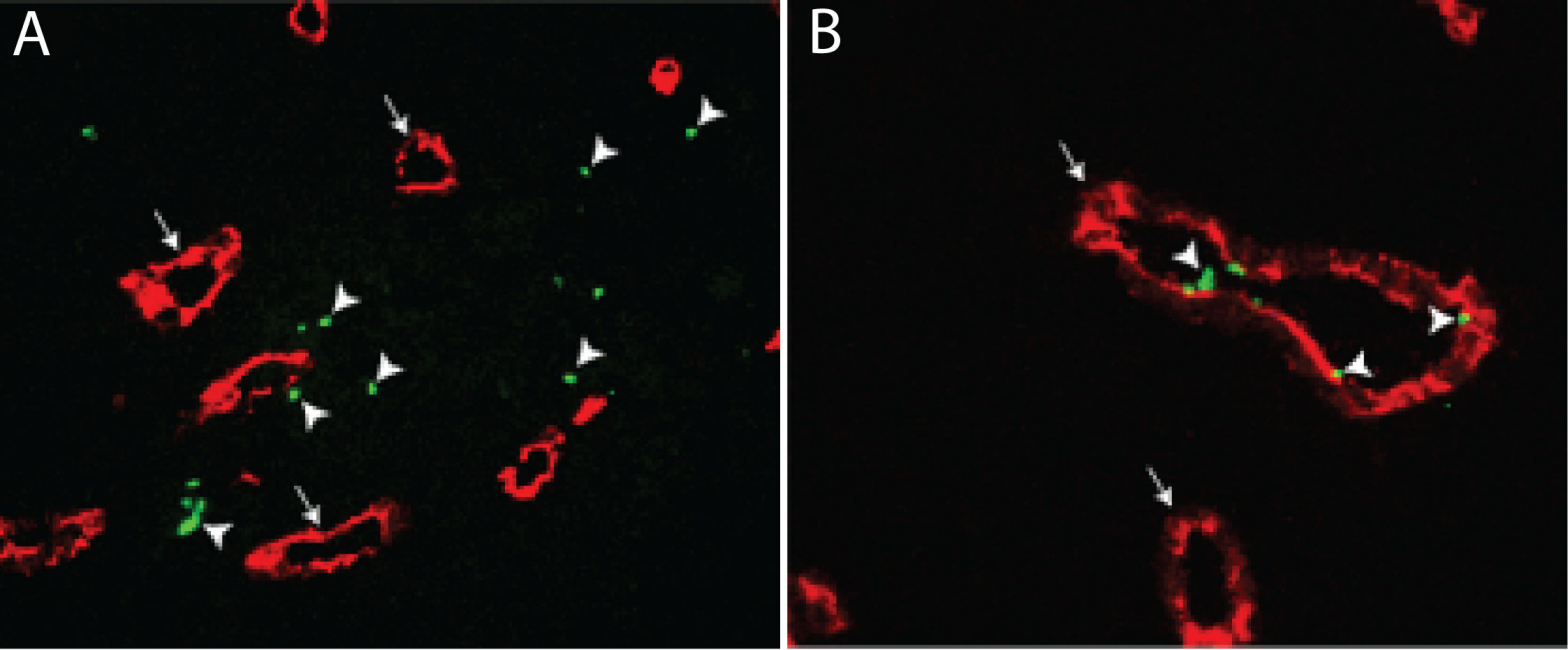
Platelet extravasation in ovarian cancer. **(A, B)** Reduced extravasation of FAK-deficient platelets into orthotopic ovarian tumors in mice. Immuno-fluorescent microscopy of tumor nodules resected from **(A)** wild-type and **(B)** platelet-specific FAK-deficient (PF4Cre; FAK^fl/fl^) mice using anti-CD31 to label endothelium (red; arrows) and anti-CD42b to label platelets (green; arrowheads). FAK-deficient platelets remain intravascular, while wild-type platelets were found to extravasate (54).

In another study on ovarian cancer, focal adhesion kinase (FAK) regulated platelet extravasation into the tumor microenvironment (**Figure 6**), with platelet-specific deletion of FAK completely preventing tumor rebound growth following cessation of antiangiogenic therapy in mice (52). As it has been reported that tumor growth may be accelerated following the cessation of antiangiogenic therapy, this study aimed to investigate the role of platelets in this phenomenon. It was found that cessation of antiangiogenic therapies such as pazopanib, bevacizumab, and the anti-VEGF antibody B20 was associated with significant tumor growth accompanied by tumor hypoxia, increased angiogenesis in the tumor microenvironment, and vascular leakage (52). This hypoxia led to increased ADP production and platelet extravasation into the tumor parenchyma, but not in mice carrying the deletion for platelet-specific FAK. In fact, simply lowering platelet counts was sufficient to significantly blunt tumor rebound following withdrawal from antiangiogenic therapy. This may result from diminished platelet release of ADP in the tumor microenvironment, which can activate the P2Y12 receptor on tumor cells and modulate the epithelial-mesenchymal transition necessary during the early stages of metastasis (53).

It has also been shown that platelet-derived transforming growth factor β1 (TGFβ1) plays a role in tumor growth, neoangiogenesis, as well as platelet extravasation into the tumor microenvironment (54). Furthermore, it was demonstrated that the prometastatic role of platelets in ovarian cancer is mediated, in a dose-dependent manner, by platelet TGFβ1, activating the Smad signaling pathway in cancer cells (55). While TGFβ1 does not entirely explain the role of extravascular platelets in ovarian cancer progression, deletion of TGFβ1 in platelets, as well as deletion of TGFβR1 on ovarian cancer cells, led to a 50% reduction of orthotopic ovarian cancer in mice (54). Platelets contain 40–100 times more TGFβ1 than any other cell; they are the primary source of circulating TBF β, and patients with ovarian cancer have elevated platelet counts and levels of serum TGFβ1 (56, 57). Platelets have also been shown to significantly increase cancer cell proliferation and migration *in vitro* (45). A critical step in metastasis is the epithelial-mesenchymal transition, a process mediated in part by direct contact between platelets and tumor cells, which results in platelet activation and increased levels of TGFβ1 activating the TGFβ1/Smad signaling pathway (55, 58, 59).

Platelets utilize a variety of G protein-coupled receptors on their surface to respond to environmental stimuli; the role of these G proteins has been studied in the context of platelet extravasation into the tumor microenvironment (60). Platelet deficiencies in Gi and G13 (but not Gq) result in a 90% reduction in platelet extravasation, and reduced tumor burden compared to control animals.

It is clear that platelet extravasation into tumor tissues plays a complex role in the tumor microenvironment and is associated with poor prognosis and aggressive tumor behavior. While work has been done to unravel the intricate interactions between platelets and tumor cells, a deeper understanding of platelet extravasation dynamics in the tumor microenvironment may be essential for developing therapies targeting cancer metastasis and improving patient outcomes.

### 2.3 The brain microvasculature

Platelets are increasingly recognized for their active role in the degeneration and regeneration processes within the central nervous system, supported by significant crosstalk between platelets and neural cells (61). Platelets contain several neurotransmitters capable of exerting effects on neurons and other cells of the brain parenchyma, and these neurotransmitters include epinephrine, dopamine, histamine, glutamate, γ-aminobutyric acid (GABA), and serotonin (17, 62, 63). In addition, platelets contain proteins such as reelin, a regulatory protein for cell migration and synaptic plasticity, brain-derived neurotrophic factor (BDNF), which plays a role in the development and function of neural circuitry, as well as vascular endothelial growth factor (VEGF) and antioxidants such as glutathione peroxidase. Aside from their intravascular functions, platelets have been reported to alter blood-brain barrier permeability and extravasate into the brain parenchyma in various conditions, including stroke, multiple sclerosis, traumatic brain injury, and demyelinating diseases (29, 64–69). These observations strongly suggest that, outside of their intravascular roles, platelets can cross the blood-brain barrier, inducing functional responses via cell-cell interactions in the parenchyma.

While studies on dermal inflammation demonstrate platelet extravasation into tissue parenchyma without apparent degranulation, studies in the brain have identified the interaction between platelets and brain-specific glycolipids after extravasation as a potent activator of platelets (26, 35, 70). Sotnikov et al. demonstrated that injection of brain-specific lipid rafts, containing sialylated gangliosides found abundantly on astroglial and neuronal cells, results in extensive systemic platelet activation and degranulation, and a life-threatening anaphylactic response in mice (70). This response was specific to brain- and spinal cord-derived lipid rafts, with those taken from other tissues failing to elicit a strong platelet response. Furthermore, the organizational structure of sialylated gangliosides on lipid rafts was required to be recognized by platelets. Of note, lipid rafts isolated from gray matter elicited a more than 2-fold higher response than those taken from white matter, and the response was specific to lipid rafts isolated from cortical neurons and astrocytes. In contrast, those isolated from oligodendrocytes did not elicit a response. These findings provide evidence for a tissue-specific activation of platelets contacting parenchymal cells in the brain.

Regulation of the blood-brain barrier is paramount to the health and maintenance of the brain. Even transient disruption of the blood-brain barrier has been shown to have marked effects on cortical tissue and neuronal health (71–73). Despite this, recent evidence reveals the presence of platelet extravasation in the cortical microvasculature. In a 2015 paper by Kazanis et al., the accumulation of platelets in the peri-ventricular region of the hippocampus was demonstrated following lysolecithin-induced demyelination in the corpus callosum (28). The extravasation of platelets into the brain parenchyma may play an important role in disease progression, as there is evidence that direct interaction with platelet CD40L in areas with evident platelet extravasation activates astrocytes and microglia in the brain during hypertension (67). Platelet extravasation in the subependymal zone of the lateral ventricular wall, a region characterized by specialized vasculature and a population of resident neural stem/progenitor cells, promoted cortical tissue maintenance and regeneration via enhanced survival of adult neural stem- and progenitor cells (28). Platelet extravasation was detected at 4 days post-lesion, and platelets accumulated not only in the lesion within the cortical vasculature and parenchymal space, but also in the subependymal zone in close proximity to proliferating neural stem/progenitor cells. Notably, an increase in blood vessel density (assessed via laminin-labeling) was reported on days 7 and 15 post-lesion. The association between extravasated platelets and neural stem cells may play an important role in wound healing post-injury, as *in vitro* exposure of platelet lysate to neural stem cells increased their cell number via enhanced cell survival and diminished apoptosis without an apparent effect on cell proliferation or differentiation potential (28). This finding is consistent with the observation of reduced cellular apoptosis and increased cell density in the subependymal zone. In the retina of mice with autoimmune-mediated encephalomyelitis, platelets have even been shown to extravasate and target gray matter, driving retinal inflammation in this disease (74).

Extravascular platelets have also been identified in the brain in a mouse model of Alzheimer’s disease. Kniewallner et al. showed that, in amyloidogenic mice, platelets were found in high numbers outside the neurovasculature and in close contact with astrocytes in the parenchyma (29). This group found platelets interact with neuronal glycolipids, leading to the secretion of platelet-derived pro-inflammatory mediators in the central nervous system. This phenomenon is worth exploring, as platelet-parenchymal interactions significantly increase the incidence and intensity of epileptic seizures in mouse models, and platelets actively secrete serotonin, enhancing blood-brain barrier permeability (27). Moreover, platelets directly stimulated neuronal electric activity and contributed to oxidative stress in neurons. This direct interaction between platelets and cells in the brain can have profound consequences. For example, intracranial injection of platelets has been shown to result in epileptic seizures comparable to those induced by injection of the GABA inhibitor pentylenetetrazol (PTZ).

Evidence of platelet extravasation in the cortical microvasculature challenges conventional views of platelet behavior within the central nervous system. Although the brain’s highly regulated microenvironment strongly regulates the low permeability of the blood-brain barrier, there is growing evidence of platelet accumulation in the brain parenchyma in the context of neuronal pathologies. While the implications of platelet extravasation in the brain have yet to be fully explored, its presence raises intriguing questions about the potential role of extravascular platelets in neuroinflammatory processes and neural repair. Further study is warranted to unravel the molecular mechanisms and functional consequences of platelet extravasation in the brain.

### 2.4 The corneal microvasculature

**Figure 7:**
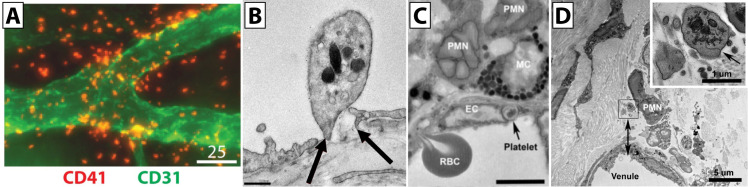
Platelet extravasation plays a crucial role in the corneal wound response. **(A)** Representative micrograph of extravascular platelets (red) around the corneal limbal vascular network (green) at 12 hours after a central corneal wound. **(B)** Representative electron micrograph of platelets in wild-type mice 8 hours after corneal abrasion. Here, an intravascular platelet can be seen positioned over a discontinuity (arrows) in the endothelium. Small gold particles (5 nm) decorate the platelet surface and denote platelet-specific CD42b immunolabeling. **(C)** Electron micrograph of a wild-type mouse cornea 8 hours after epithelial abrasion showing a platelet and a red blood cell in the process of traversing the inflamed endothelium of a limbal venule. Extravascular polymorphonuclear cells (PMNs) are in contact with a perivascular mast cell (MC). **(D)** Blood cell extravasation 8 hours after corneal abrasion in wild-type mice. Electron micrograph reveals an extravascular PMN and several extravascular platelets next to a venule. The extravascular platelets can be seen in the inset box to the immediate left of the PMN, and the lower edge of the inset box is positioned 6 µm from the luminal endothelial surface of the venule (double arrowhead). An enlarged view of the inset (upper right) shows an extravascular platelet identified by a surface-connected canaliculus (arrow). A second extravascular platelet is located in the lower left. Panel **(A)** scale bar = 25 µm. Scale bar in panel **(B)** = 0.5 µm. Scale bar in panel **(C)** = 60 µm. Panel **(D)** scale bar = 5 µm. Panel **(D)** inset image scale bar = 1 µm. Adapted from De La Cruz et al., *Int J Mol Sci* 20021;22(14) and Hargrave et al., *PLoS One* 2020;15(9):e0238750. CC BY 4.0. Changes were made (33, 75).

The cornea is an avascular tissue, with blood vessels relegated to the corneal limbus at its periphery. Despite this, blood cells and limbal vasculature play vital roles in the health of this tissue. Studies from the authors’ laboratory show that abrasion of the central cornea, though it does not directly injure the blood vessels in the limbus, results in the recruitment and extravasation of platelets in the limbal vasculature, and deficiencies in this response result in an impaired wound healing process (32, 33, 75, 76) (**Figure 7**). In the corneal limbus, platelet extravasation occurs exclusively in venules with no apparent extravasation in arterioles (33). Furthermore, platelet extravasation corresponds with neutrophil extravasation, peaking at 12 hours post-injury. In this mouse model of corneal injury, P-selectin is critically important as mice deficient in P-selectin show reduced platelet and neutrophil extravasation, which was restored by infusion of platelets from wild-type mice (32, 77). Moreover, platelet extravasation also requires leukocyte β2 integrin, as evidenced in mice expressing low levels of CD18 (CD18hypo mice), which show normal levels of neutrophil extravasation but a marked reduction in platelet extravasation (32).

As platelets extravasate and interact with tissue parenchyma, they degranulate and release growth factors into the limbus and peripheral cornea. Stromal cells known as keratocytes are affected by growth factors that can promote their differentiation into fibroblast and myofibroblast phenotypes. Transforming growth factor (TGF)-β and platelet-derived growth factor (PDGF), found within α-granules, effectively promote keratocyte differentiation, while insulin-like growth factor-1 (IGF-1), TGF-β, and PDGF are known to enhance keratocyte DNA synthesis *in vitro* (78–83). *In vivo*, following a corneal abrasion, stromal keratocytes lying beneath the abraded epithelium die, and the recovery of stromal keratocyte density is dependent on platelet extravasation (32). Moreover, extravasated platelets are a rich source of vascular endothelial growth factor (VEGF), enhancing acute corneal epithelial wound closure and nerve regeneration post-injury in mice (77). Finally, the observation that perivascular limbal macrophages phagocytose extravascular platelets raises the possibility that the macrophage phenotype can be altered, given that phagocytosis of platelets by monocytes is known to promote their differentiation into proinflammatory macrophages (33, 84, 85).

While platelet extravasation at the corneal limbus has been demonstrated, their method of extravasation is less clear. While it is conceivable that platelets attach to neutrophils and are carried across the vessel wall during neutrophil diapedesis, ultrastructural observations of the injured cornea do not support this mechanism. Indeed, while neutrophils appear to pass through endothelial pores ranging in size from 0.3 to 2 μm, platelets pass through sub-micron pores independent of any attachment to neutrophils (33). In the cornea abrasion model, platelet extravasation is also linked to mast cell degranulation, which results in venule engorgement. Platelets are observed to translocate through small endothelial pores in the engorged venules where vascular pressures are elevated. The importance of that engorgement is supported by observations in CD18hypo mice and mast cell-deficient mice, which exhibit significant reductions in venule engorgement alongside marked reductions in platelet extravasation.

While the mechanism(s) by which platelets extravasate in the cornea require further study, platelets were shown to play a significant role in corneal wound healing. Platelet extravasation is required for proper and timely wound healing following injury and plays a role in fibroblast and myofibroblast differentiation, in addition to macrophage polarization, and may play a role in nerve regeneration following injury (32, 33, 75–77, 81, 85). Understanding the mechanisms that govern platelet extravasation in the limbal vasculature may hold promise for the development of targeted therapies enhancing corneal wound healing or mitigating conditions that threaten patient’s vision.

### 2.5 The lung microvasculature

The microvasculature of the lung serves a critical role as the body’s interface for gas exchange and immune surveillance, making it susceptible to various pathological conditions, including infection, inflammation, and vascular injury. While platelets play a traditional hemostatic role in the lung microvasculature, they have also been shown to participate in lung immune response and tissue repair mechanisms. For example, platelets exhibit a protective effect against pulmonary edema, enhance the pulmonary inflammatory response, and assist in pathogen clearance (86, 87). While platelets play a role in lung protection, they also mediate damage in acute lung injury and acute respiratory distress syndrome. Indeed, granzyme B-dependent platelet-induced apoptosis has been demonstrated in the lungs of septic mice (88). In addition to the known roles of platelets in health and disease, there is a growing body of literature reporting the presence of extravascular platelets in lung tissue and the alveolar space (89–94). Activated platelets can release bioactive molecules involved in immune modulation and tissue repair, such as Interleukin-1β (IL-1β) and tumor necrosis factor-α (TNF-α), which amplify local inflammatory responses and promote the recruitment of immune cells to sites of injury or inflammation (95–97). Furthermore, extravascular platelets may release growth factors and chemokines that stimulate fibroblast proliferation and collagen deposition and promote fibroblast transition into myofibroblasts (98, 99). While acute inflammation can contribute to tissue remodeling and repair, excessive or chronic accumulation of platelets can lead to aberrant fibrosis and impaired lung function (100).

**Figure 8:**
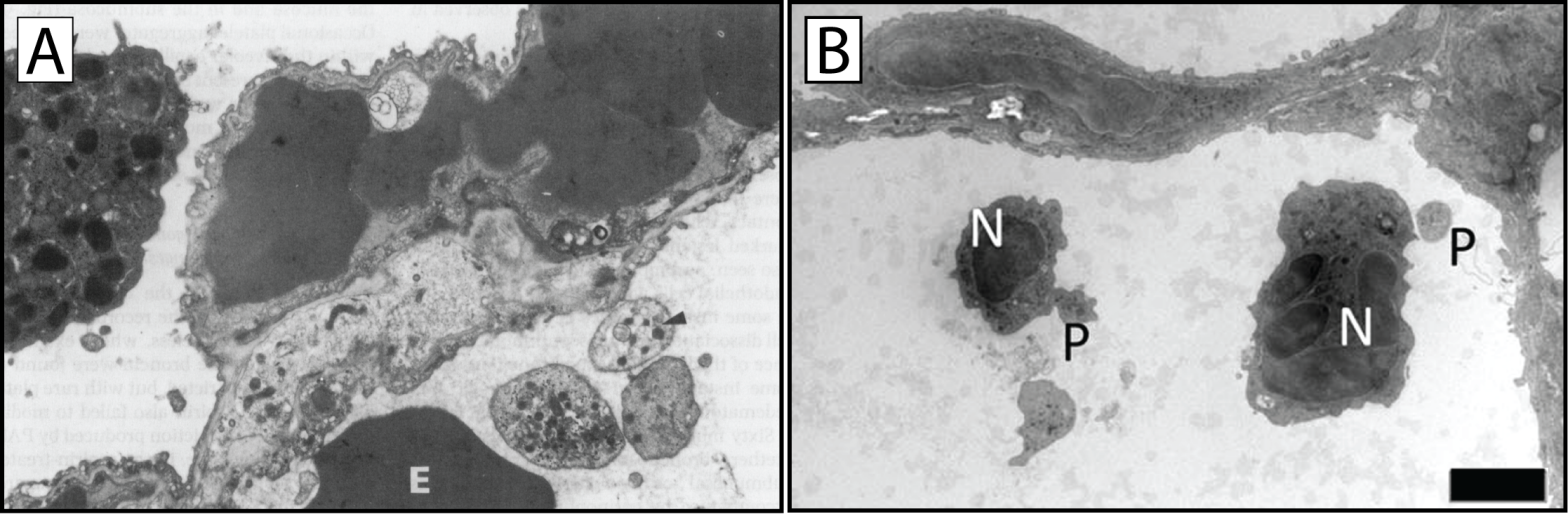
Electron microscopy evidence of platelet extravasation into the alveolar space. **(A)** An electron micrograph of lung tissue experiencing capillary stasis 3 minutes after PAF-acether injection. Extravascular platelets (arrowhead) can be seen in the alveolar space alongside an erythrocyte (E) and an eosinophil (arrow); x6000 magnification. **(B)** Bacterial pneumonia was induced in wild-type mice, and ultrathin sections of lung tissue were imaged via transmission electron microscopy. Platelets (P) and neutrophils (N) are located in the alveolar space during recovery from pulmonary inflammation. Zoom in panel A = 6000x; Panel B scale bar = 2 µm. Adapted from Rossaint et al., *J Exp Med* 2021;218:e20201353. CC BY-NC-SA 4.0. Changes were made; and with permission from Lellouch-Tubiana et al., *Br J Exp Pathol* 1985;66:345-355. ^©^ Wiley (101, 102).

Dysregulated platelet extravasation in the lung has been implicated in a number of respiratory diseases, including pulmonary fibrosis, asthma, and pneumonia (97). In lung disorders with a fibrotic component, persistent platelet accumulation may contribute to excessive collagen deposition and scar formation, restricting lung inflation and tissue perfusion. In asthma, platelets exacerbate airway inflammation and bronchoconstriction, aggravating respiratory symptoms. Moreover, in infectious lung diseases such as pneumonia, platelet extravasation facilitates pathogen clearance but may also exacerbate lung injury and inflammation, highlighting the complex role of platelets within the lung microenvironment (89, 97). Though the direct effects of intravascular and extravasated platelets in the lung still require disentanglement, there is solid evidence of extravasated platelets in lung parenchymal tissue and the alveolar space through imaging and lung gavage studies (**Figure 8**) (89, 97, 101, 102). While the roles of extravasated platelets still require further elucidation, a greater understanding of the drivers and molecular mechanisms of platelet extravasation in the lung microvasculature may provide novel insights into lung disease and the identification of potential therapeutic targets for intervention.

### 2.6 The liver microvasculature

**Figure 9:**
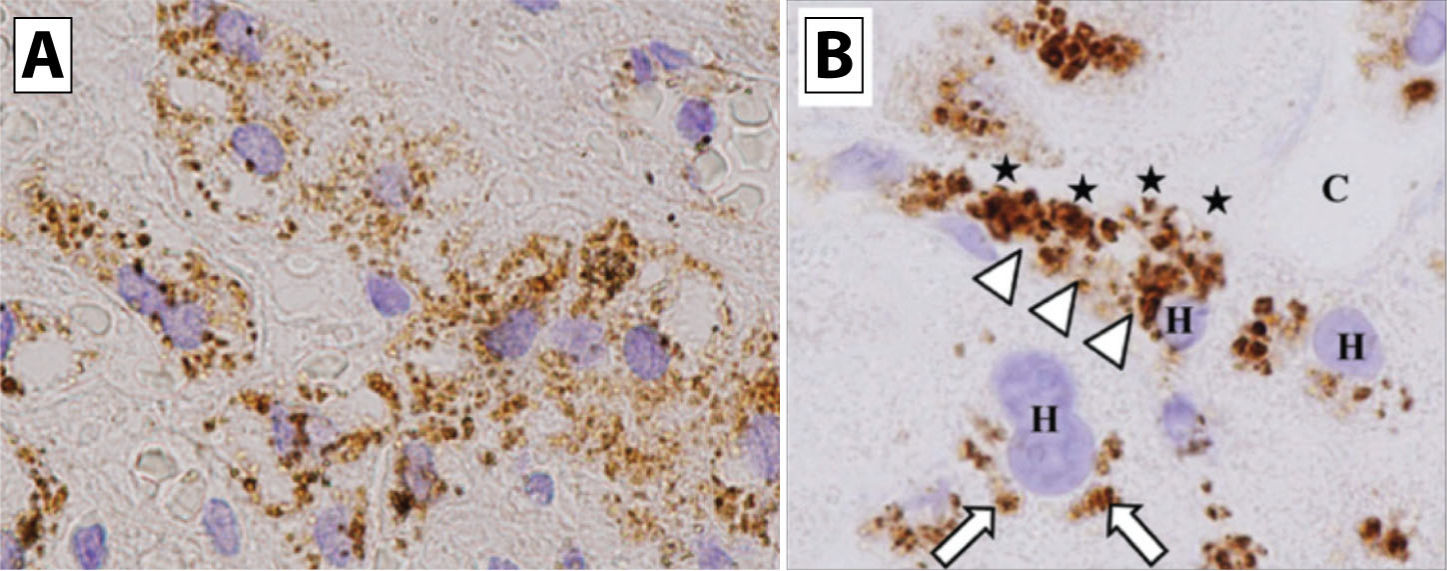
Platelet extravasation in recipients of liver transplantation and patients who received oxaliplatin-based chemotherapy. **(A)** Immunohistochemical CD42b staining in hepatic tissue resected following preoperative oxaliplatin chemotherapy. CD42b staining was observed around the central vein, and in contact with hepatocytes, in zone 3, where hepatocyte destruction was observed. CD42b was present as aggregates attached to hepatocytes along the sinusoid. **(B)** Immunohistochemical staining of CD42b in liver allograft tissue. Liver biopsy was taken on postoperative day 91, where CD42b staining was noted in zone 3 as aggregates attached to hepatocytes (arrowheads) along the sinusoid (stars) and in the hepatocyte cytoplasm (arrows). C, central vein; H, hepatocyte. Magnification in panel B is x1000. Adapted with permission from Nakanuma et al., *Exp Ther Med* 2015;9:1119–1124 and Tajima et al., *Mol Clin Oncol* 2015;3:555-558. ^©^ Spandidos Publications (31, 110).

The liver microvasculature begins at the portal venules, which become terminal portal venules before reaching the sinusoidal network, followed by post-capillary terminal hepatic venules, collecting venules, and muscular venules. The hepatic acinus, containing a terminal portal venule at its center and terminal hepatic venules in the periphery, can be divided into three zones emanating from the portal venule. The first zone contains vessels with narrow lumens, large endothelial fenestrations, and large numbers of Kupffer cells. In contrast, the second and third zones contain larger lumens with smaller fenestrations and lower Kupffer cell numbers (103). While platelets mediate hepatic regeneration by entering the space of Disse and interacting with hepatocytes, they have also been shown to participate in liver pathology by extravasating into the liver parenchyma and modulating the immune and inflammatory responses (104–107). Oxaliplatin-based chemotherapy, while playing a key role in the treatment of patients with colorectal liver metastasis, has been associated with hepatic sinusoidal obstruction syndrome. This syndrome is characterized by hepatic sinusoidal dilation, hepatocyte atrophy, perisinusoidal fibrosis, and nodular regenerative hyperplasia resulting in liver dysfunction (108). In recipients of liver transplantation who develop a thrombotic microangiopathy-like disorder or sinusoidal obstruction syndrome, as well as patients who received oxaliplatin-based chemotherapy, platelet extravasation and accumulation in zone three has been noted with platelets in contact with hepatocytes and within the spaces of Disse (31, 51, 109, 110) (**Figure 9**). These platelets were shown to secrete various factors such as platelet-activating factor (PAF), thromboxane A2 (TXA2), thrombospondin, vascular endothelial growth factor (VEGF), and plasminogen activator inhibitor-1 (PAI-1), all of which are thought to contribute to liver injury (111). In addition, PAF and TXA2 are believed to cause central vein occlusion and portal hypertension, while transforming growth factor-β (TGF-β), which is activated by thrombospondin, can cause perisinusoidal collagen deposition and diminish substance exchange in the space of Disse (112, 113). Further, PAI-1 and TGF-β can inhibit hepatocyte growth factors and interfere with liver regeneration (114). Additional case studies have reported extravasated platelet aggregation in liver zone 3 correlates with sinusoidal obstruction syndrome and thrombotic microangiopathy-like disorder following liver transplantation (109, 110, 115). Interestingly, platelets have also been found within the space of Disse at 24 hours after induction of sepsis in a lipopolysaccharide (LPS)-induced mouse model associated with the formation of neutrophil extracellular traps (NETs), detachment of liver sinusoidal endothelial cells, and early sepsis-induced hepatic dysfunction (116, 117).

The liver is a highly vascular tissue and can be particularly susceptible to the effects of dysregulated platelet responses. Platelet extravasation into the space of Disse and liver parenchyma may play an important but understudied role in liver pathology. As such, exploring the mechanisms responsible for platelet extravasation in the liver and targeting the platelet-mediated responses of liver parenchymal cells may hold promises for the development of novel therapies aimed at improving the lives of patients suffering liver-related complications.

## 3 Mechanisms of extravasation

**Figure 10:**
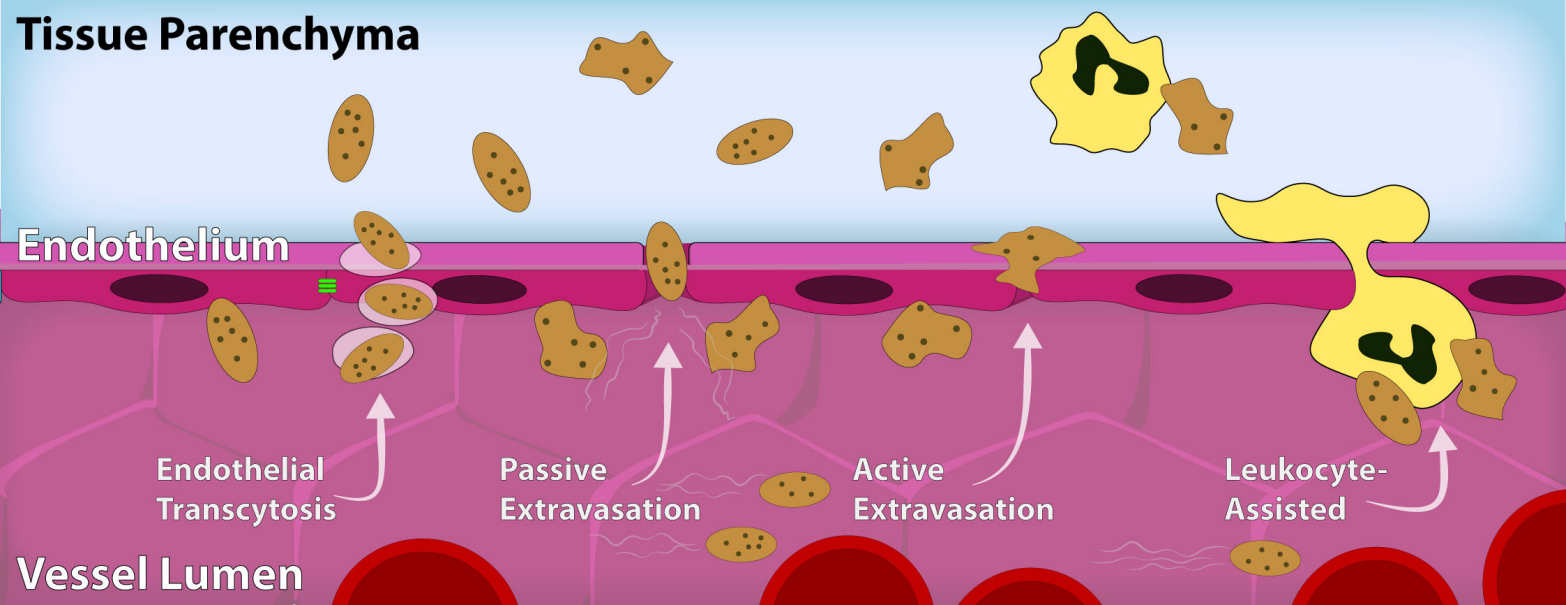
Proposed models of platelet extravasation into tissue parenchyma. While the mechanism by which platelet extravasation occurs may vary between vascular beds and conditions, four main mechanisms have been proposed: endothelial transcytosis, passive extravasation, active extravasation, and leukocyte-assisted extravasation. Platelets (brown) have been shown to undergo endothelial transcytosis without disruption of endothelial junctional proteins or extensive activation and degranulation of platelets. It has also been theorized that platelets may extravasate through discontinuities in the vascular endothelium via a pressure-based mechanism. Alternatively, some literature postulates that platelets have the capacity for limited motility and may be able to undergo active extravasation into the parenchymal space. It has also been postulated that platelets “piggyback” on leukocytes (yellow) via a leukocyte-assisted process as they escape the vessel lumen.

While evidence exists for platelet extravasation in a host of tissues and pathological states, the mechanisms by which platelets extravasate out of the vasculature and into tissue parenchyma are not well explored. However, based on available data, platelet extravasation may occur through multiple, potentially overlapping pathways. Here we describe four possible mechanisms in the literature (**Figure 10**) (1): endothelial transcytosis of platelets through the vascular endothelium, (2) passive extravasation dependent upon pressure and cytoskeletal dynamics, (3) active extravasation dependent upon the concept of platelet motility, and (4) leukocyte-assisted extravasation in which platelet-leukocyte aggregates exit the vasculature together. Some of these mechanisms are supported by direct evidence, such as endothelial transcytosis and passive platelet extravasation, whereas others are inferred from indirect evidence or extrapolated from existing leukocyte trafficking paradigms, such as active platelet extravasation and leukocyte-assisted extravasation. While the mechanism of extravasation may differ from tissue to tissue, or by the nature of the disease or disorder, a deeper understanding of the mechanisms responsible for platelet extravasation may open the door for a more nuanced exploration of this phenomenon, as well as identify novel therapeutic pathways for treating several human afflictions.

### 3.1 Endothelial transcytosis of platelets

Cellular extravasation through undamaged endothelium can occur through either the paracellular or transcellular pathways (118–122). The precise requirements for both routes of extravasation vary from vascular bed to vascular bed, with the barrier properties of vessels depending upon several factors. For example, the barrier properties of vessels vary according to structural differences such as the presence of continuous, fenestrated, or sinusoidal endothelium, the state of junctional proteins between endothelial cells, the presence and characteristics of the endothelial glycocalyx, as well as the health of the vessels and surrounding parenchyma (120, 123, 124). Paracellular migration requires transient disruption of cell-cell junctions, which are comprised of transmembrane proteins with extracellular domains and intracellular partners bound to their cytoplasmic tails (125). It is critical, however, that this process be tightly regulated because excessive transmigration of cells at inappropriate locations can result in tissue injury or the development of inflammatory disorders such as atherosclerosis, chronic inflammation, and rheumatoid arthritis (125). While the paracellular route has long been considered the primary route for extravasation from the vasculature, there now exists a wealth of evidence for the transcellular migration of cells (transcytosis), including leukocytes and platelets, even in tissues with extremely effective barrier properties such as the blood-brain barrier (26, 121, 126). As such, the evidence supports the ability for endothelial cells to internalize platelets under inflammatory conditions, supporting a vesicular transport mechanism.

Aside from the work of Feng and Dvorak, the use of the transcellular pathway by platelets has been relatively unexplored (26). However, the mechanisms by which leukocytes utilize transcytosis to extravasate have been well explored (121, 127). Once attached to the vascular endothelium, leukocytes probe the endothelial surface with invadosome-like protrusions in a process known as tenertaxis (127–129). These invadosome-like protrusions make contact with regions of the endothelial membrane that are enriched in Cav1, plasmalemmal vesicle-associated protein-1 (PLVAP-1), and SNARE proteins. Cells lacking these molecules have been shown to be incapable of forming a transcellular pore, resulting in inefficient transcytosis (121, 126). This interaction can result in the formation of “transmigratory cups” enriched in intercellular adhesion molecule (ICAM)-1 and lymphocyte function-associated antigen (LFA)-1 and the recruitment and fusion of endothelial vesicles and membrane surface. In fact, the deletion of ICAM-1 and ICAM-2 removes the capacity for leukocyte transcytosis in the brain (130). These regions become enriched in endothelial vesicles and vesiculo-vacuolar organelles (VVOs), which can span the entire thickness of vascular endothelial cells (131). Individual vesicular elements that form VVOs have an average diameter of 108 ± 32 nm and can fuse to form transendothelial channels ranging from 30–150 nm in diameter, and transcellular channels can be rich in ICAM-1 and caveolin-1 (132, 133). This interaction, and subsequent formation of transendothelial channels, allows leukocytes to pass through the vascular endothelium without disrupting the endothelial junctions (122). Given the close intravascular and extravascular relationship between leukocytes and platelets, and the role of ICAM-1 in platelet-endothelial interactions, it is possible that platelets utilize similar methods for transcytosis through the vascular endothelium (134). The mechanism of VVO fusion to create a transendothelial channel is thought to be mediated by the release of VEGF from leukocytes (135, 136). This is important to note, as platelets are also a source of VEGF in circulation and they release VEGF upon aggregation (137). Furthermore, many tumors express VEGF, resulting in increased vascularization in the tumor microenvironment and higher levels of VEGF within platelets, as well as an increased level of platelet extravasation (138, 139). While these similarities between leukocyte and platelet capabilities are intriguing, whether platelets utilize a similar transmigratory mechanism as leukocytes requires further study.

### 3.2 Active extravasation

While platelets are canonically considered non-motile, they contain many essential elements required for migration. Despite their lack of a nucleus and limited protein synthesis capabilities, they contain the cytoskeletal and enzymatic mechanisms required for cell motility, they produce matrix metalloproteinases necessary for extracellular matrix degradation, and they express cellular adhesion proteins alongside chemokine receptors (140, 141). While studies on platelet motility are limited, one report suggests that bacterial cleavage products can initiate the movement of platelets (142). *In vitro* studies have reported that certain bacterial cleavage products, such as formyl peptides, can induce platelet migration at a speed of 13.07 ± 1.10 µm/min. Furthermore, platelets have been reported to migrate *in vitro* towards a collagen substrate in a factor XI-dependent manner (143). Interestingly, collagen-induced platelet chemotaxis did not require direct platelet-collagen contact, suggesting a fundamental difference between the requirements for platelet activation and platelet chemotaxis (144). One report indicates that plasma enzymes may cleave collagen, thus establishing a chemical gradient for platelet chemotaxis (143). However, the concept of platelet motility, especially in the context of extravasation, requires further exploration.

Whether platelets actively transmigrate through the endothelium in a manner analogous to leukocyte diapedesis remains unclear. While platelets express adhesion proteins capable of firm arrest on the vascular surface, in addition to mechanisms for cytoskeletal remodeling, direct evidence of active platelet extravasation is missing from the literature. As such, active platelet extravasation should be considered as a proposed mechanism requiring further experimental validation.

### 3.3 Passive extravasation of platelets

There are a number of mechanisms by which a pore or endothelial discontinuity can develop in the vascular endothelium. Once a pore is present, macromolecules and cells have the potential to pass through into the extravascular space (145). Electron microscopy has provided direct evidence of red blood cell (RBC) extravasation through pores in the vascular endothelium (33, 146). It is important to note that RBCs lack robust machinery for cell motility and rely on pressure gradients to extravasate through the vessel wall. While platelets contain extensive machinery for cell motility, evident in their ability to alter their shape upon activation, their ability to navigate the vascular wall and extravascular space can be most appropriately compared to that of RBCs. Though platelet and RBC extravasation can occur following leukocyte diapedesis, they can also cross through sub-micron endothelial discontinuities (33, 35, 146, 147).

The ability of blood cells to exit passively through sub-micron endothelial discontinuities rests on their cellular deformability. It was hypothesized early on that RBCs, dependent upon cellular stiffness, may pass through endothelial pores based on hydrostatic pressure gradients (146). While platelets are much smaller than RBCs, they are less deformable (148, 149) because of their cortical microtubule ring (**Figure 1**) and the properties of their actin cytoskeleton (150). Despite this, both RBCs and platelets have been found outside undamaged vasculature, primarily extravasating from engorged venules utilizing sub-micron pores where elevated vascular pressures are present (33, 151). In further studies utilizing mast cell-deficient mice, where there is a marked reduction in venule engorgement, RBC and platelet extravasation were significantly diminished, consistent with the hydrostatic pressure-driven hypothesis of extravasation. While a pressure-based, passive mechanism for platelet extravasation is intriguing, further studies must determine how platelets might traverse the discontinuous endothelium without sticking within pores to create “plugs,” as previously reported (152). Passive extravasation likely occurs in settings where increased vascular permeability exists, such as sepsis, tumor-associated angiogenesis, and acute inflammation. In these contexts, platelet extravasation into the tissue parenchyma likely correlates with endothelial barrier disruption rather than with evidence of an active extravasation process.

### 3.4 Leukocyte-assisted platelet extravasation

**Figure 11:**
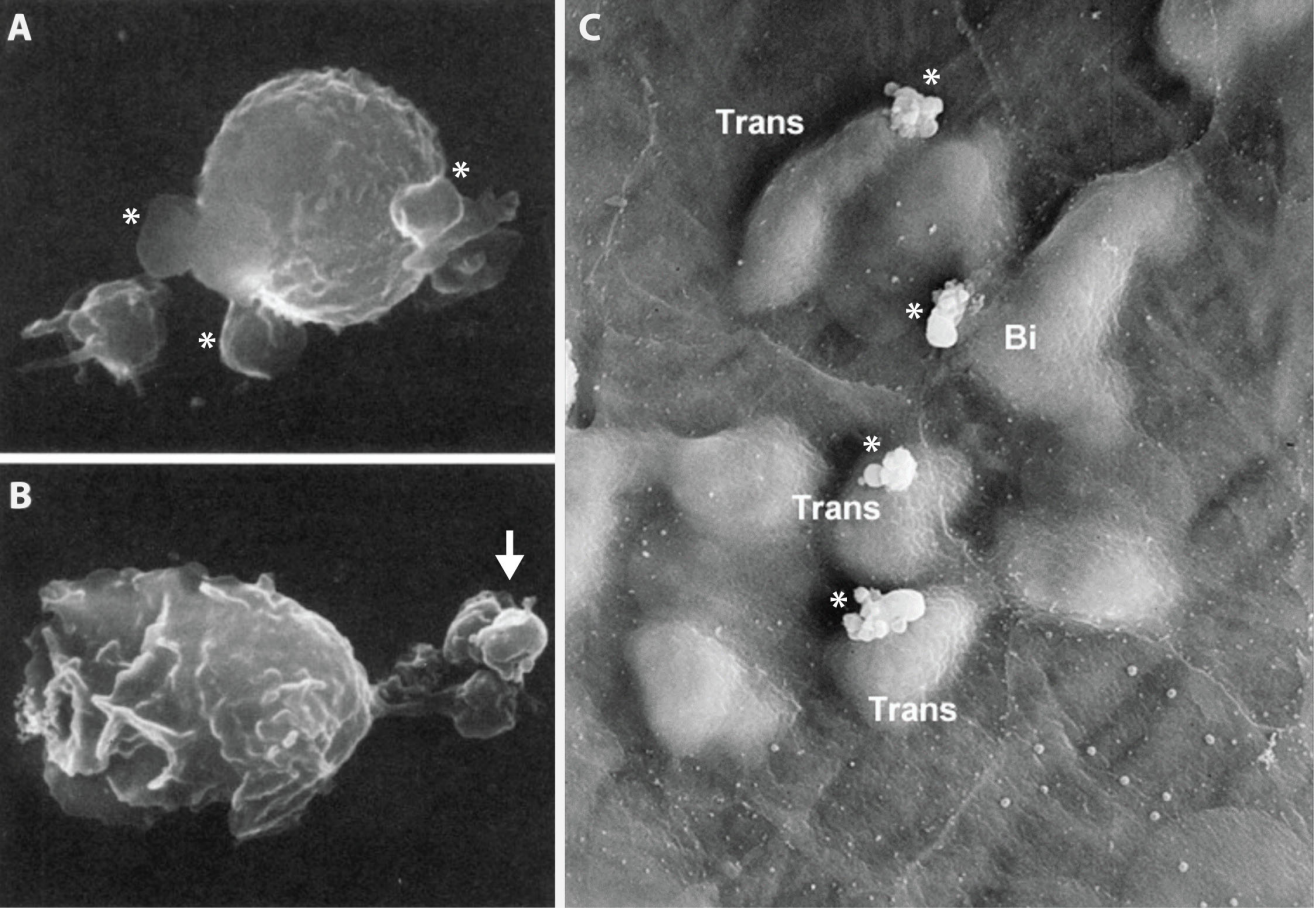
Platelets are relegated to the uropod of migrating neutrophils and may be left behind during extravasation. **(A, B)** Binding of thrombin-activated canine platelets to resting and chemotactically stimulated canine neutrophils imaged via scanning electron microscopy. **(A)** Platelets (asterisks) are randomly distributed on the surface of unstimulated neutrophils. **(B)** Upon chemotactic stimulation, platelets become clustered at the uropod (arrow) of traveling neutrophils. **(C)** Transmigrating PMNs and platelets in the mouse lip vasculature 15 minutes after injection of MIP-2, viewed from the vessel lumen. PMNs were found to extravasate at bicellular junctions (Bi) in addition to transcellular (Trans) migration through the body of the endothelial cells. While PMNs passed the endothelium into the tissue parenchyma, platelets were found stuck to the luminal endothelium (asterisks), failing to extravasate with associated PMNs. Panel A & B magnification = x10,000. Adapted from *Blood*, Vol. 87, Doré M, Burns AR, Hughes BJ, Entman ML, Smith CW, Chemoattractant-Induced Changes in Surface Expression and Redistribution of a Functional Ligand for P-Selectin on Neutrophils, pp. 2029–2037, Copyright 1996, with permission from Elsevier (156).

One potential route for platelet extravasation can be described as “leukocyte-assisted extravasation.” This mechanism leans on the close relationship between platelets and leukocytes in response to injury or infection. The interaction between platelets and leukocytes is necessary for an effective response to vascular injury, as it facilitates clot formation, immune signaling, and the recruitment of immune cells to sites of injury or inflammation. Platelet-neutrophil aggregates can form within the blood circulation or on the vascular endothelium (153). Furthermore, it has been demonstrated that platelets play an active role in guiding leukocytes, such as neutrophils, to their sites of extravasation (151, 154). Platelet interaction with neutrophils is facilitated by the binding of P-selectin or GPIbα to PSGL-1 or Mac-1 on neutrophils, respectively (155). Upon initial platelet attachment to neutrophils, platelets are distributed randomly on the neutrophil surface (156). However, shortly (within 30 seconds) after the introduction of chemotactic factors, neutrophils begin to change shape, with the leading edge of the cell taking on a ruffled appearance and the trailing end forming what is known as a uropod. Upon undergoing this shape change, the organization of organelles, cytoskeleton, and surface proteins becomes compartmentalized between the leading edge of the cell and the uropod (157). While the leading edge contains a host of chemosensory receptors and integrins for cell migration, the uropod holds organelles, including the endoplasmic reticulum, Golgi apparatus, and mitochondria, and surface proteins including CD43, CD44, ICAM-1, -2, and -3, in addition to PSGL-1 (158). With the reorganization of PSGL-1 to the uropod, the once random placement of platelets becomes redistributed to the uropod of the migrating cells (**Figure 11**) (156). As these migrating neutrophils begin translocating through the vascular endothelium, platelets may be pulled through the vascular endothelium while attached to the transmigrating neutrophil’s uropod. However, direct support for this mechanism is lacking. Moreover, given the close surface membrane associations between the migrating neutrophil and the endothelial pore, the platelets could be stripped off the neutrophil and left behind on the endothelial surface (**Figure 11**). While the mechanistic concept of platelets “piggybacking” on leukocytes to exit the vasculature is not substantially supported by evidence in the literature, it does seem that platelet extravasation occurs at sites supporting active leukocyte extravasation (35).

### 3.5 Inter-mechanistic relationships and tissue-specific considerations

Platelet extravasation mechanisms are unlikely to be mutually exclusive. In an environment of increased vascular permeability, passive platelet extravasation may be facilitated while simultaneously enhancing leukocyte recruitment, thereby promoting leukocyte-assisted platelet extravasation as well. Similarly, enhanced platelet adhesion to inflamed endothelium may precede either endothelial transcytosis of platelets or subsequent detachment and extravasation into the extravascular space. Extravasation of leukocytes who have formed platelet-leukocyte aggregates may result in leukocyte-assisted platelet extravasation in addition to subsequent platelet deposition on, and transcytosis through, associated endothelial cells. Tissue-specific vascular architecture may result in bias toward specific mechanisms. For example, tissues characterized by continuous, tightly regulated endothelial barriers, such as the central nervous system, may require receptor-mediated interactions to initiate platelet extravasation, whereas fenestrated vascular beds that are more permeable may permit more passive platelet extravasation. Mast cells, whose role in platelet extravasation were highlighted in the corneal section, show tissue-specific differences in their perivascular densities. Moreover, mast cell activation is regulated by autocrine and paracrine tissue-specific signaling which regulates their contribution to modulating vascular permeability, leukocyte recruitment, and platelet activation (159). As such, mast cells may play a role in platelet extravasation to a varying degree from tissue to tissue.

The dominant triggers for platelet extravasation will vary by tissue and disease contexts. In the tumor microenvironment, or in the context of chronic inflammation, angiogenic remodeling and chronic endothelial activation may favor sustained platelet recruitment while acute or systemic inflammation may promote platelet accumulation driven by alterations in vascular permeability. Platelet extravasation likely exists along a mechanistic spectrum defined by tissue-specific architecture, endothelial activation, vascular permeability, leukocyte recruitment, and the activation status of platelets. Taken collectively, the evidence supporting a role for platelet extravasation suggests that, despite mechanistic through-lines such as requiring CD18, P-selectin, and the presence of mast cells, platelet extravasation is not a uniform phenomenon but rather a context-dependent process shaped by vascular architecture, inflammatory environment, and tissue-specific cellular interactions.

## 4 Therapeutic and translational implications of platelet extravasation

Recognizing the ability of platelets to extravasate into tissues and alter the tissue microenvironment further expands their role beyond hemostasis and thrombosis as active participants in immune regulation and disease response. Platelet extravasation as a possible novel point of intervention may open up the potential for new therapeutic strategies in a host of disease processes. While currently approved anti-platelet therapies were developed to prevent thrombosis, their effects on tissue platelet accumulation via extravasation remain incompletely characterized. A rich context-dependent mechanistic understanding of platelet extravasation may open up therapeutic avenues wherein pathological platelet-tissue interactions can be down- or up-regulated selectively while preserving their essential hemostatic function. While established anti-platelet agents, such as cyclooxygenase inhibitors, P2Y12 receptor antagonists, and αIIbβ3 inhibitors, effectively reduce platelet activation and aggregation within the vasculature, their effect on platelet extravasation and accumulation within the extravascular space is not well understood. Given that anti-platelet therapies can influence inflammatory biomarkers and disease progression it may be possible that modulatory effects on platelet extravasation contribute to these responses. However, given the need to thread the needle between clotting and bleeding risks when targeting platelets, a more selective strategy targeting platelet extravasation as a novel intervention point may enable the disruption of pathological platelet-tissue interactions without global suppression of platelet activity.

Along this line, therapeutics targeting specific platelet-cell interactions, such as P-selectin-PSGL-1 interaction or platelet-leukocyte aggregation, have the potential to alter platelet extravasation dynamics without completely diminishing their hemostatic capabilities. These therapeutic strategies could be especially relevant in chronic inflammatory diseases, tumor malignancy, and pathologies where platelet accumulation is correlated with disease severity. Of course, the therapeutic potential for targeting platelet extravasation is likely to be tissue- and disease-dependent. In the context of cancer, targeting extravasated platelets may decrease immune suppression and slow cancer progression, while in the context of neuroinflammatory disease it may have the potential to mitigate neurovascular injury or prolong disease onset. Conversely, in the context of sterile injury or infectious disease, diminishing platelet extravasation may blunt repair mechanisms and host defense. The context-dependent roles of extravasated platelets highlights the importance of disease- and tissue-specific evaluation of targeted therapeutics. Furthermore, translational studies will present several challenges. First, separating the intravascular and extravascular effects of platelets in complex disease processes will necessitate creative study design. Second, redundant adhesion and platelet-cell interaction pathways may complicate therapeutics aimed at their selective targeting. Finally, altering platelet function carries the risk of tipping hemostasis into the realms of bleeding or thrombotic disorder. Future studies will need to delineate novel therapeutic techniques that alter platelet extravasation mechanics while sparing their beneficial hemostatic response.

## 5 Conclusions

Our ever-expanding understanding of platelet functions beyond their typical role in hemostasis and thrombosis underscores their importance in the body’s response to damage and disease. The presence of extravasated platelets, once relegated to a product of vascular trauma and bleeding, is beginning to be recognized as a common and essential occurrence, implicating platelets in a variety of physiological and pathological processes. It has become increasingly apparent that platelets exert multiple effects beyond their intravascular functions and untangling the intricate relationship between extravascular platelets and the cells and molecules within tissue microenvironments will be crucial for understanding a host of pathophysiological processes. In essence, platelet extravasation and its interaction with blood cells, the vascular endothelium, and cells of the parenchyma in different tissues under physiological and pathological conditions represent a scientific frontier ripe for investigation. Moving forward, it will be important to better define the mechanisms by which platelets extravasate into tissues, as well as to increase our understanding of the roles and functions that extravascular platelets play in health and disease. Ultimately, further study of this important and complex process holds the potential to unravel its complexity and improve our understanding of the multifaceted roles platelets play and may provide the key to developing novel therapeutic strategies across a spectrum of diseases.
